# Supplementary material for: Genome-Wide Identification and Characterization of the PP2C Family from Zea mays and Its Role in Long-Distance Signaling
Source: Plants (Basel). 2023 Sep 1;12(17):3153. doi: 10.3390/plants12173153 (PMC10490008; doi:10.3390/plants12173153)
Supplement: Supplementary file 1 [file plants-12-03153-s001.zip › plants-2525558-supplementary.pdf]

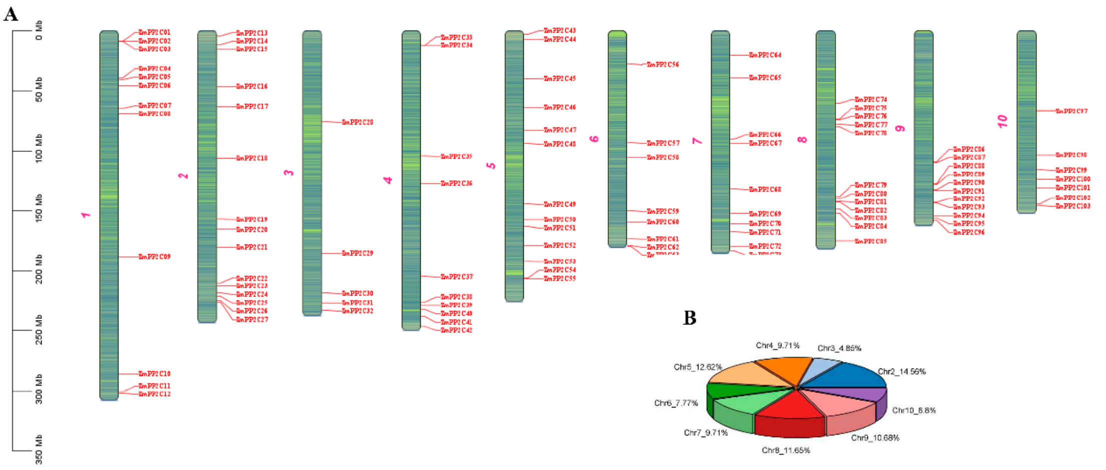

Figure S1. Chromosome distribution of *ZmPP2C* genes on 10 chromosomes of maize. (A) The size of each chromosome and its corresponding *ZmPP2Cs* distribution, with the chromosome number is indicated at the left of each chromosome. The scale bar on the left indicates the size of the chromosomes; (B) The pie chart shows the percentage of *ZmPP2Cs* on each chromosome.

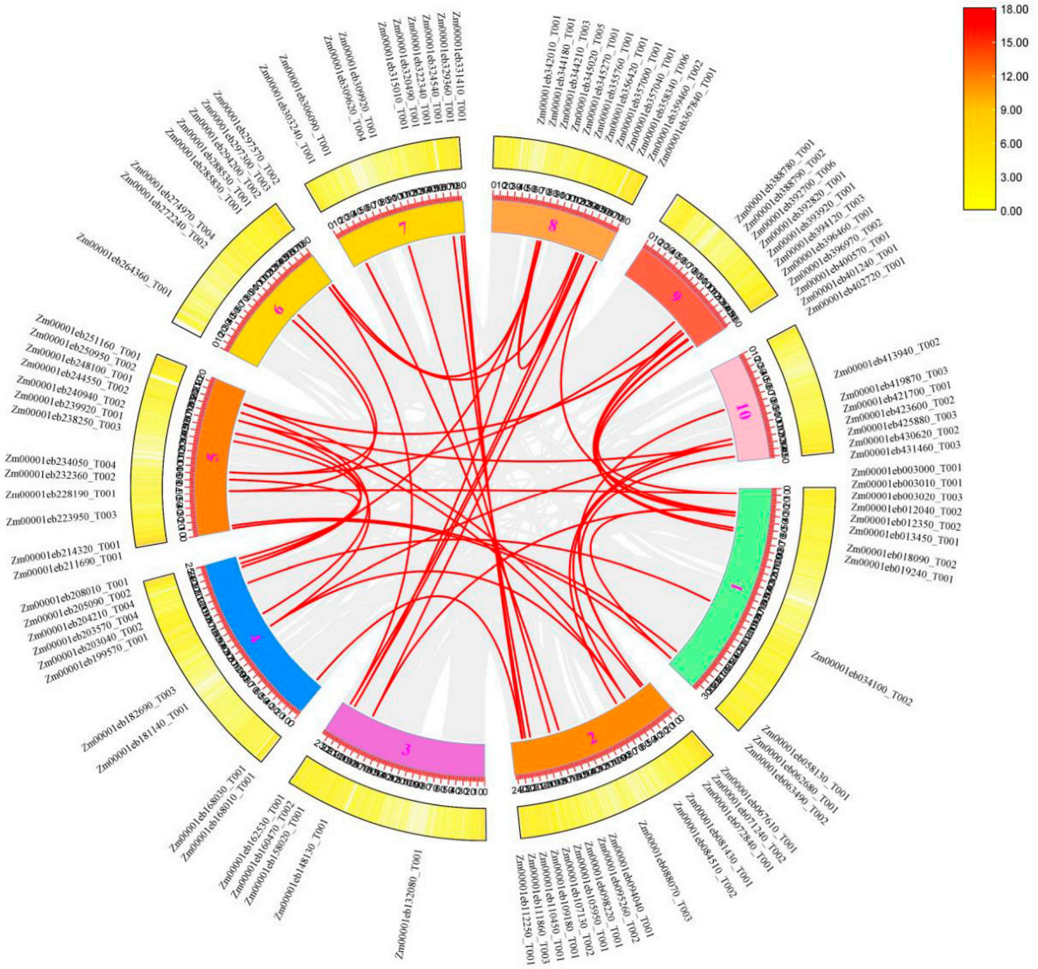

Figure S2. Syntenic analysis of the *ZmPP2Cs*. The colored box represents the chromosome, the line indicates the position of the syntenic gene pair on the chromosome, the red line denoted the *ZmPP2C* gene pairs, while the gray line shows other syntenic gene pairs in the maize.

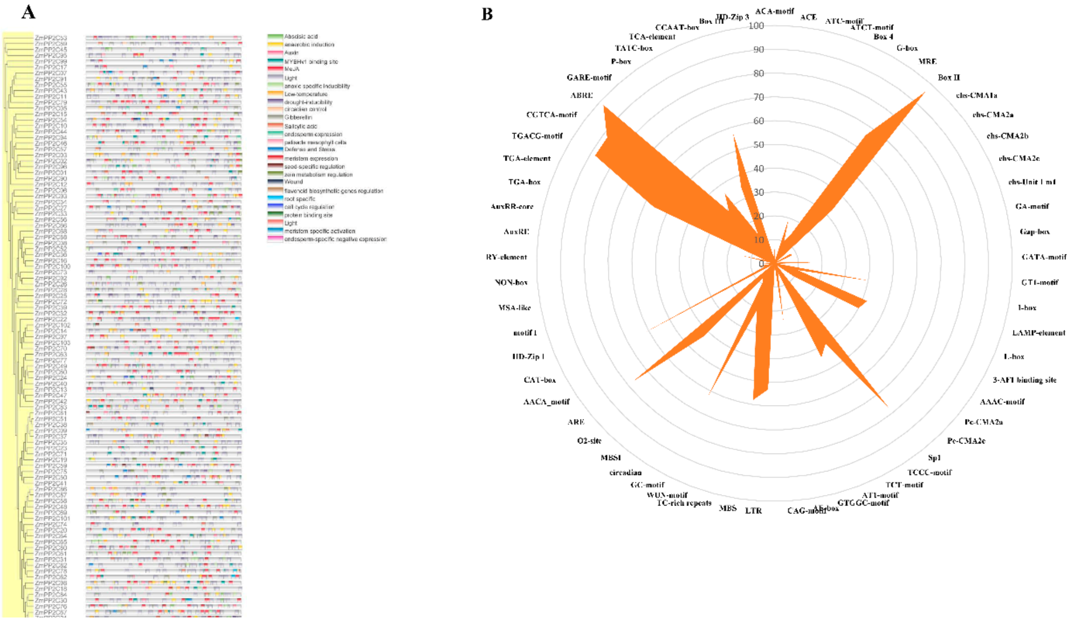

Figure S3. Statistical analysis of cis-elements in the promoter of *ZmPP2C* genes. **(A)** Cis-element analysis of the promoter regions of *ZmPP2C* genes. Differently colored shapes represent different elements. (B) Proportions of different cis-elements in *ZmPP2C* genes.

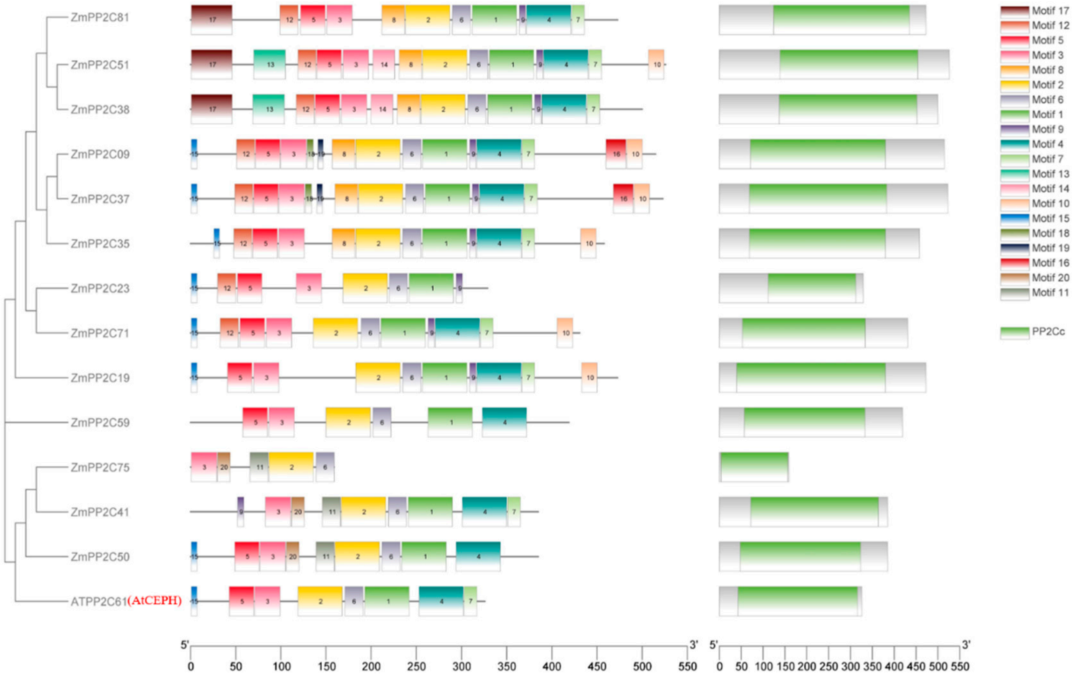

Figure. S4. Evolutionary tree, motif, domain, and cis-elements of 13 *ZmPP2C* genes and *AtCEPH*. The length of each protein can be estimated using the scale at the bottom.

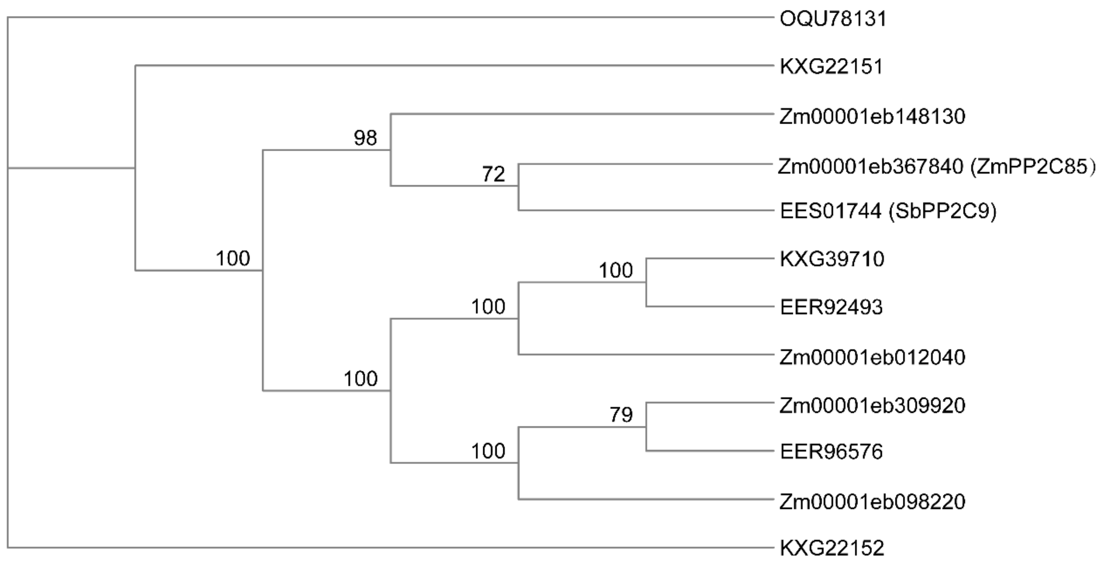

Figure S5. The evolutionary tree was constructed using TBtool software.

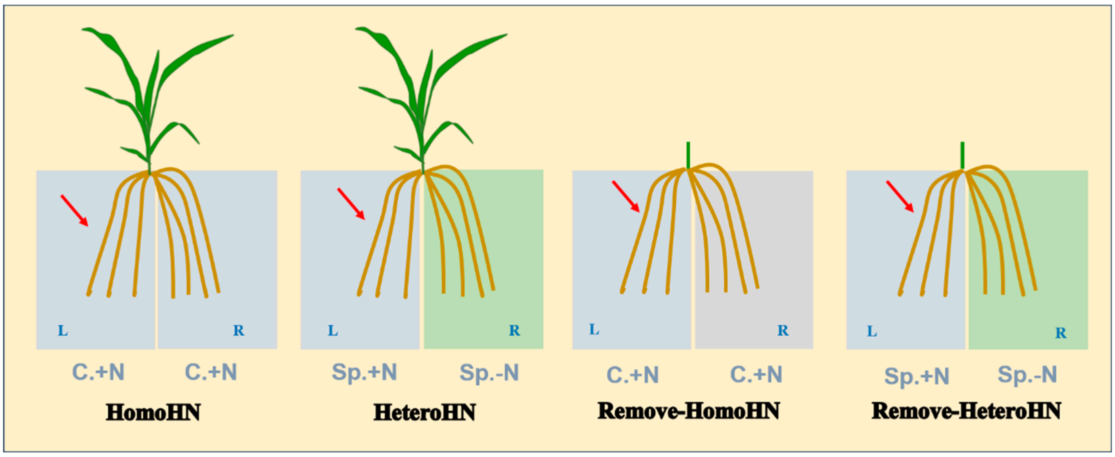

Figure S6. Split-root system in which the root system of a plant is separated into left (L) and right (R) parts that are exposed to different nutrient conditions. The red arrow points to the sampled tissue.

Table S1. Properties and predicted locations of ZmPP2C proteins.

Gene_name	Chr	Chr location	Chain	Number of amino	Instability index	Theoretical pI	Molecular weight (kDa)	Subcellular location

				acids				
ZmPP2C01	1	8216041-8220640	+	357	45.9	7.7	39.95	Nucleus.
ZmPP2C02	1	8222270-8226801	-	399	48.18	8.47	44.16	Nucleus.
ZmPP2C03	1	8237814-8243635	+	399	48.16	8.69	44.07	Nucleus.
ZmPP2C04	1	39067264-39069360	-	396	61.22	6.43	42.04	Nucleus.
ZmPP2C05	1	40463650-40466683	+	740	51.38	7.62	81.28	Nucleus.
ZmPP2C06	1	45160373-45162447	+	388	60.55	6.18	40.79	Nucleus.
ZmPP2C07	1	64567706-64576354	+	962	51.43	6.04	106.3	Nucleus.
ZmPP2C08	1	69007071-69011416	+	440	40.37	5.67	47.36	Nucleus.
ZmPP2C09	1	188239653-188244354	-	515	42.56	5.1	55.05	Nucleus.
ZmPP2C10	1	285863639-285867090	-	376	40.05	9.66	41.02	Nucleus.
ZmPP2C11	1	300484734-300488297	-	615	49.22	5.99	66.29	Chloroplast.
ZmPP2C12	1	302449509-302453159	-	379	37.54	6.83	41.05	Nucleus.
ZmPP2C13	2	4263315-4267702	+	284	33.89	4.46	30.66	Nucleus.
ZmPP2C14	2	11409181-11413754	+	315	40.17	8.66	34.16	Nucleus.
ZmPP2C15	2	15309005-15312211	+	392	44.13	6.63	43.69	Chloroplast.
ZmPP2C16	2	46187668-46192641	+	444	50.22	5.98	47.36	Nucleus.
ZmPP2C17	2	62682531-62686631	+	521	44.28	4.62	57.48	Chloroplast.
ZmPP2C18	2	106146629-106150713	+	457	62.3	5.71	49.04	Nucleus.
ZmPP2C19	2	156948040	-	473	41.99	5	51.02	Chloroplast

		-156950830						.
ZmPP2C20	2	165213344-165220565	-	581	52.69	6.65	63.72	Chloroplast.
ZmPP2C21	2	180274627-180276009	+	350	62	6.77	36.96	Nucleus.
ZmPP2C22	2	209737398-209739063	+	306	42.08	6.45	33.35	Nucleus.
ZmPP2C23	2	212637710-212640699	-	329	46.68	8.96	35.9	Cell membrane.
ZmPP2C24	2	217993093-217995851	+	146	44.13	4.64	16.26	Nucleus.
ZmPP2C25	2	220861770-220866133	-	454	47.05	4.82	47.5	Chloroplast.
ZmPP2C26	2	224728063-224731366	+	429	44.46	7.94	46.1	Nucleus.
ZmPP2C27	2	226054557-226057199	+	429	45.08	7.37	44.57	Chloroplast.
ZmPP2C28	3	75762675-75779291	-	484	42.94	7.66	51.52	Nucleus.
ZmPP2C29	3	185093948-185095922	-	408	58.76	5.92	43.28	Nucleus.
ZmPP2C30	3	218456535-218462539	-	410	48.54	6.11	43.44	Nucleus.
ZmPP2C31	3	226455074-226460821	+	484	44.71	4.48	50.7	Chloroplast.
ZmPP2C32	3	232736476-232740529	+	384	39.44	5.29	41.19	Chloroplast.
ZmPP2C33	4	11862186-11864645	+	436	50.66	6.71	45.46	Chloroplast.
ZmPP2C34	4	11872182-11874449	+	106	26.24	7.03	11.07	Nucleus.

ZmPP2C35	4	104502063-104516614	-	458	38.13	6.66	49.97	Chloroplast.
ZmPP2C36	4	127220133-127245164	-	449	58.85	6.41	48.22	Nucleus.
ZmPP2C37	4	204429970-204434309	-	523	41.37	4.89	56.37	Nucleus.
ZmPP2C38	4	225770293-225784317	+	500	36.5	7.94	53.21	Chloroplast.
ZmPP2C39	4	228511195-228533871	-	1084	38.98	4.77	120.5	Cell membrane.
ZmPP2C40	4	232221731-232231065	+	357	38.35	5.97	38.55	Chloroplast.
ZmPP2C41	4	237957702-237960413	-	385	43.76	6.76	41	Nucleus.
ZmPP2C42	4	246114806-246118717	-	290	26.65	4.52	31.21	Nucleus.
ZmPP2C43	5	2629765-2633632	+	632	51.5	5.4	68.21	Chloroplast.
ZmPP2C44	5	6892027-6894932	+	367	44.89	9.69	40.09	Nucleus.
ZmPP2C45	5	39903133-39909872	+	594	49.88	4.24	61.92	Nucleus.
ZmPP2C46	5	63655085-63659727	-	394	49.15	8.88	43.8	Nucleus.
ZmPP2C47	5	82332663-82336140	+	348	31.45	4.66	37.39	Chloroplast.
ZmPP2C48	5	93913340-93920865	+	359	33.13	5.05	39.23	Nucleus.
ZmPP2C49	5	144347259-144352230	-	365	39.31	6.07	39.3	Chloroplast.
ZmPP2C50	5	157163131-157165949	-	385	44.68	6.76	41.17	Nucleus.
ZmPP2C51	5	163143385-	-	526	37.45	7.11	55.8	Chloroplast.

		163148831						
ZmPP2C52	5	178759459-178763854	-	470	57.89	5.92	50.8	Nucleus.
ZmPP2C53	5	191858823-191861109	-	322	36.08	7.94	34.33	Chloroplast.
ZmPP2C54	5	205611839-205614878	-	387	41.09	8.9	43.11	Chloroplast.
ZmPP2C55	5	206230718-206241041	+	550	51.69	5.73	62.08	Chloroplast.
ZmPP2C56	6	27476632-27483313	-	431	44.19	7.44	45.09	Chloroplast.
ZmPP2C57	6	93300348-93306754	+	390	50.55	9.04	43.15	Nucleus.
ZmPP2C58	6	105423342-105428237	+	365	35.36	4.78	40.08	Nucleus.
ZmPP2C59	6	149955406-149959124	+	419	49.46	8.89	45.53	Mitochondrion.
ZmPP2C60	6	159266327-159276528	+	679	53.11	10.34	75.59	Chloroplast.
ZmPP2C61	6	173043093-173047120	-	370	39.53	5.08	39.72	Nucleus.
ZmPP2C62	6	178866102-178869300	+	429	47	4.85	45.4	Chloroplast.
ZmPP2C63	6	179555644-179563259	+	499	51.48	4.48	54.31	Chloroplast.
ZmPP2C64	7	19919587-19933729	+	583	51.52	7.43	63.74	Chloroplast.
ZmPP2C65	7	39226238-39227878	+	239	39.97	6.36	25.82	Mitochondrion.
ZmPP2C66	7	90311008-90325606	-	360	39.03	7.03	39.03	Nucleus.
ZmPP2C67	7	93421327-93422962	+	358	53.91	6.82	37.6	Nucleus.
ZmPP2C68	7	131676591	-	431	46.58	6.4	47.16	Chloroplast

		-131681994						.
ZmPP2C69	7	151898541-151904632	+	363	37.21	4.72	39.54	Nucleus.
ZmPP2C70	7	160529252-160538878	-	290	28.63	6.75	31.74	Nucleus.
ZmPP2C71	7	167218410-167222438	-	431	44.42	5.92	46.36	Chloroplast.
ZmPP2C72	7	179460272-179463998	-	352	38.57	4.78	37.24	Mitochondrion.
ZmPP2C73	7	183528521-183532791	+	429	41.95	7.6	46.03	Nucleus.
ZmPP2C74	8	60211518-60221459	+	255	35.43	6.26	28.08	Nucleus.
ZmPP2C75	8	73727431-73727910	-	159	43.23	4.44	16.94	Nucleus.
ZmPP2C76	8	73966393-73969285	-	406	63.66	8.57	43.44	Nucleus.
ZmPP2C77	8	77866011-77878684	-	505	47.96	4.42	55	Chloroplast.
ZmPP2C78	8	79568849-79573001	+	423	44.71	4.84	44.9	Nucleus.
ZmPP2C79	8	137523807-137525931	-	508	47.15	6.33	54.39	Nucleus.
ZmPP2C80	8	139606577-139630785	+	391	37.54	5.53	41.77	Nucleus.
ZmPP2C81	8	142212025-142217746	+	473	39.87	8.79	50.38	Chloroplast.
ZmPP2C82	8	142405510-142412923	-	440	39.56	4.66	46.57	Chloroplast.
ZmPP2C83	8	148115539-148123566	+	375	45.85	7.29	40.48	Nucleus.
ZmPP2C84	8	151777850-	+	394	60	6.61	41.71	Nucleus.

		151782663						
ZmPP2C85	8	175000529-175002418	-	413	52.84	6.76	43.82	Nucleus.
ZmPP2C86	9	109385970-109391305	-	365	34.61	4.67	40.21	Nucleus
ZmPP2C87	9	109598658-109603762	-	366	37.93	4.47	40.15	Nucleus.
ZmPP2C88	9	127296654-127301563	+	446	38.21	5.25	48	Nucleus.
ZmPP2C89	9	127653234-127654184	-	316	31.62	6.29	33.69	Chloroplast.
ZmPP2C90	9	132025472-132029930	+	393	45	8.48	43.6	Chloroplast.
ZmPP2C91	9	132988328-132996488	-	1008	47.09	6.02	111.16	Nucleus.
ZmPP2C92	9	142637006-142640717	+	430	38.07	6.95	45.92	Chloroplast.
ZmPP2C93	9	143668232-143670483	-	388	60.19	6.01	40.95	Chloroplast.
ZmPP2C94	9	153924323-153927090	-	391	49.92	7.93	42.15	Nucleus.
ZmPP2C95	9	155700197-155705998	-	596	36.94	4.07	61.39	Nucleus.
ZmPP2C96	9	158229333-158233704	+	399	44.13	8.69	44.15	Nucleus.
ZmPP2C97	10	66549934-66564838	-	336	59.54	6.37	36.53	Nucleus.
ZmPP2C98	10	103573069-103578268	+	464	63.69	5.26	48.83	Chloroplast.
ZmPP2C99	10	115576486-	-	521	43.55	4.66	57.18	Nucleus.

		115581534						
ZmPP2C100	10	123473400 - 123478449	-	443	49.44	5.98	47.54	Cell membrane.
ZmPP2C101	10	130813394 - 130818703	+	365	40.32	6.42	39.86	Nucleus.
ZmPP2C102	10	143862049 - 143866289	-	318	40.93	8.66	34.56	Nucleus.
ZmPP2C103	10	145825189 - 145837613	-	284	29.15	6.16	31.2	Nucleus.

Table S2. Estimates of the divergence style for maize's duplicated PP2C paralogs

**Gene1**	**Gene2**	**Ka**	**Ks**	**Ka_Ks**	**Selection pressure**
ZmPP2C05	ZmPP2C11	0.423919646	1.096443202	0.386631652	Purifying selection
ZmPP2C04	ZmPP2C29	0.302249727	0.806245259	0.374885587	Purifying selection
ZmPP2C09	ZmPP2C37	0.074529355	0.371648009	0.20053748	Purifying selection
ZmPP2C10	ZmPP2C44	0.038169012	0.277091725	0.137748653	Purifying selection
ZmPP2C11	ZmPP2C43	0.044313058	0.229026555	0.193484369	Purifying selection
ZmPP2C01	ZmPP2C46	0.174363156	2.135489862	0.081650191	Purifying selection
ZmPP2C04	ZmPP2C85	0.304972935	0.78456759	0.388714674	Purifying selection
ZmPP2C01	ZmPP2C96	0.027201814	0.18570984	0.146474815	Purifying selection
ZmPP2C08	ZmPP2C88	0.024936569	0.141858508	0.175784796	Purifying selection
ZmPP2C05	Zm00001eb397680_T001	0.145729926	0.271879609	0.536009033	Purifying selection
ZmPP2C06	ZmPP2C93	0.034702102	0.288058781	0.120468822	Purifying selection
ZmPP2C07	ZmPP2C91	0.037790493	0.161115712	0.234554982	Purifying selection
ZmPP2C97	ZmPP2C14	0.142214563	1.381400977	0.102949517	Purifying selection

ZmPP2C102	ZmPP2C14	0.035989971	0.165081486	0.218013372	Purifying selection
ZmPP2C99	ZmPP2C17	0.019814263	0.151310838	0.130950717	Purifying selection
ZmPP2C100	ZmPP2C16	0.028716548	0.17558755	0.163545469	Purifying selection
ZmPP2C100	ZmPP2C36	0.201263243	1.10619562	0.181941818	Purifying selection
ZmPP2C100	ZmPP2C52	0.203224846	1.090057336	0.186435006	Purifying selection
ZmPP2C27	ZmPP2C33	0.091305647	0.269341099	0.33899634	Purifying selection
ZmPP2C16	ZmPP2C36	0.192169148	1.121249844	0.171388339	Purifying selection
ZmPP2C15	ZmPP2C54	0.149788736	0.937284477	0.159811391	Purifying selection
ZmPP2C16	ZmPP2C52	0.208102211	1.056224945	0.197024518	Purifying selection
Zm00001eb107120	ZmPP2C71	0.127211179	0.497846226	0.255523036	Purifying selection
ZmPP2C25	ZmPP2C72	0.069486265	0.260214915	0.267034137	Purifying selection
ZmPP2C26	ZmPP2C73	0.02001699	0.183831163	0.108887904	Purifying selection
ZmPP2C21	ZmPP2C67	0.058137202	0.20573101	0.282588424	Purifying selection
ZmPP2C20	ZmPP2C64	0.050940059	0.200919447	0.253534739	Purifying selection
ZmPP2C31	ZmPP2C61	0.290339744	1.364172008	0.21283221	Purifying selection
ZmPP2C29	ZmPP2C85	0.03762902	0.175694989	0.214172415	Purifying selection
ZmPP2C30	ZmPP2C76	0.254145745	0.729474364	0.348395719	Purifying selection
ZmPP2C30	ZmPP2C84	0.065456544	0.20849435	0.313948764	Purifying selection
ZmPP2C31	ZmPP2C82	0.032159823	0.185013593	0.173824112	Purifying selection
ZmPP2C36	ZmPP2C52	0.02653372	0.178502849	0.148645919	Purifying selection
Zm00001eb185410	ZmPP2C53	0.456261657	0.727032902	0.627566725	Purifying selection
ZmPP2C40	ZmPP2C49	0.027115185	0.221153838	0.122607798	Purifying selection

ZmPP2C41	ZmPP2C50	0.173175001	0.339671375	0.509831013	Purifying selection
ZmPP2C42	ZmPP2C47	0.022167219	0.189352473	0.117068546	Purifying selection
ZmPP2C40	ZmPP2C77	0.274283391	1.482834647	0.184972338	Purifying selection
ZmPP2C48	ZmPP2C58	0.118292946	1.039633759	0.113783287	Purifying selection
ZmPP2C46	ZmPP2C57	0.015385155	0.18509215	0.083121596	Purifying selection
ZmPP2C54	ZmPP2C90	0.3933866	2.639743615	0.149024548	Purifying selection
ZmPP2C55	ZmPP2C91	0.44617599	2.385674954	0.187022959	Purifying selection
ZmPP2C48	ZmPP2C86	0.116475057	0.947829191	0.122886126	Purifying selection
ZmPP2C62	ZmPP2C78	0.048739116	0.181881644	0.267971607	Purifying selection
ZmPP2C63	ZmPP2C77	0.061202395	0.226204474	0.270562265	Purifying selection
ZmPP2C61	ZmPP2C80	0.058141994	0.340544188	0.170732598	Purifying selection
ZmPP2C76	ZmPP2C84	0.253277957	0.789100001	0.320970671	Purifying selection
ZmPP2C77	ZmPP2C83	0.210999515	0.917678039	0.229927606	Purifying selection

Table S3. Functionally annotated cis-elements identified in the promoters of 13 *ZmPP2Cs*

Cis-Element	Functions of Cis-Elements	Number of Genes
ACA-motif	light responsiveness	6
ACE	light responsiveness	1
ATC-motif	light responsiveness	2
ATCT-motif	light responsiveness	19
Box 4	light responsiveness	10
G-box	light responsiveness	14
MRE	light responsiveness	66
Box II	light responsive	96
chs-CMA1a	light responsive	33
chs-CMA2a	light responsive	1
chs-CMA2b	light responsive	8
chs-CMA2c	light responsive	8
chs-Unit 1 m1	light responsive	1

GA-motif	light responsive	1
Gap-box	light responsive	5
GATA-motif	light responsive	15
GT1-motif	light responsive	1
I-box	light responsive	40
LAMP-element	light responsive	10
L-box	light responsive	42
3-AF1 binding site	light responsive	40
AAAC-motif	light responsive	10
Pc-CMA2a	light responsive	1
Pc-CMA2c	light responsive	2
Sp1	light responsive	78
TCCC-motif	light responsive	40
TCT-motif	light responsive	44
AT1-motif	light responsive	4
GTGGC-motif	light responsive	7
AE-box	light response	24
CAG-motif	light response	1
LTR	low-temperature responsiveness	53
MBS	drought-inducibility	58
TC-rich repeats	defense and stress responsiveness	31
WUN-motif	wound-responsive	21
GC-motif	anoxic specific inducibility	62
circadian	circadian control	9
MBSI	flavonoid biosynthetic genes regulation	8
O2-site	zein metabolism regulation	43
ARE	anaerobic induction	77
AACA_motif	endosperm-specific negative expression	1
CAT-box	meristem expression	61
HD-Zip 1	palisade mesophyll cells	7
motif I	root specific	4
MSA-like	cell cycle regulation	8
NON-box	meristem specific activation	1
RY-element	seed-specific regulation	11
AuxRE	auxin-responsive	1
AuxRR-core	auxin responsiveness	14
TGA-box	auxin-responsive	4
TGA-element	auxin-responsive element	56
TGACG-motif	MeJA-responsiveness	88
CGTCA-motif	MeJA-responsiveness	88
ABRE	abscisic acid responsiveness	98
GARE-motif	gibberellin-responsive	29
P-box	gibberellin-responsive	36
TATC-box	gibberellin-responsiveness	14

TCA-element	salicylic acid responsiveness	35
CCAAT-box	MYBHv1 binding site	57
Box III	protein binding site	4
HD-Zip 3	protein binding site	3

Table S4. The primers for qRT-PCR.

Gene name	Primer name	primer sequence（5'-3')	Product length (bp)
ZmPP2C19	RT-ZmPP2C19-F1	GCCTAAACCTGACGAAGATG	101
	RT-ZmPP2C19-R1	ACTTGTCGAAGGAATCCAAAT	
ZmPP2C37	RT-ZmPP2C37-F-3	CAGGGATTGGACCTTGTAAT	93
	RT-ZmPP2C37-R-3	GAGTTGCAGAGCAGTTAGAT	
ZmPP2C41	RT-ZmPP2C41-F-1	CAGGAACTGTCCTACCAATTAC	94
	RT-ZmPP2C41-R-1	GTGGTTGCTGTGCATTTATC	
ZmPP2C50	RT-ZmPP2C50-F-1	GAACGTGGTGTACGTGATG	156
	RT-ZmPP2C50-R-1	AATCCCTGGCAGAGTATGA	
ZmPP2C54	RT-ZmPP2C54-F-1	GACCTCAGGAAAGTTGAGAAG	101
	RT-ZmPP2C54-R-1	GTATCCCTCTCTTGCAGTAATC	
ZmPP2C69	RT-ZmPP2C69-F-1	TGCCAACCTCTCTCCATAAA	92
	RT-ZmPP2C69-R-1	CTTATCTCCCAGCTCAGTTAGT	
ZmPP2C71	RT-ZmPP2C71-F4	GCAGAACGGATCAAGAAGT	142
	RT-ZmPP2C71-R4	CATAATCCTTGAGGCAGAAGT	
ZmPP2C75	RT-ZmPP2C75-F-2	GTTCGAGACCATGGACAG	166
	RT-ZmPP2C75-R-2	CTTCAGGTAGCACGTCTC	
ZmPPC81	RT-ZmPP2C81-F-1	GAACACAGGGTTGAAGATACT	121
	RT-ZmPP2C81-R-1	GATCCCTGTCCATCACATAAA	
ZmPP2C85	RT-ZmPP2C85-F-1	CTGCGAAGAAGCCAAGTC	140
	RT-ZmPP2C85-R-1	GGAACCTCCTCATCTCCAA	
ZmElF1	ZmElF1-F	GCCGCCAAGAAGAAATGATGC	220
	ZmElF1-R	CGCCAAAAGGAGAAATACAAG	
